# Analysis of Microbial Water Contamination, Soil Microbial Community Structure, and Soil Respiration in a Collaborative First-Year Students as Scholars Program (SAS)

**DOI:** 10.3389/fmicb.2020.590035

**Published:** 2020-12-17

**Authors:** Leah T. Stiemsma, Stephen D. Davis, Jay L. Brewster

**Affiliations:** Natural Science Division, Pepperdine University, Malibu, CA, United States

**Keywords:** first-year seminar, microbiome, plant physiology, ecology, undergraduate research

## Abstract

The persistence of college students in STEM majors after their first-year of college is approximately 50%, with underrepresented populations displaying even higher rates of departure. For many undergraduates, their first-year in college is defined by large class sizes, poor access to research faculty, and minimal standing in communities of scholars. Pepperdine University and Whittier College, funded by a National Science Foundation award to Improve Undergraduate Stem Education (NSF IUSE), partnered in the development of first-year classes specifically geared to improve student persistence in STEM and academic success. This Students as Scholars Program (SAS) engaged first-year undergraduates in scholarly efforts during their first semester in college with a careful approach to original research design and mentoring by both faculty and upperclassmen experienced in research. Courses began by introducing hypothesis formulation and experimental design partnered with the scientific focus of each course (ecological, biochemical, microbiological). Students split into research teams, explored the primary literature, designed research projects, and executed experiments over a 6–7 week period, collecting, analyzing, and interpreting data. Microbiology-specific projects included partnerships with local park managers to assess water quality and microbial coliform contamination at specified locations in a coastal watershed. In addition, students explored the impact of soil salinity on microbial community structure. Analysis of these samples included next-generation sequencing and microbiome compositional analysis via collaboration with students from an upper division microbiology course. This cross-course collaboration facilitated additional student mentoring opportunities between upperclassmen and first-year students. This approach provided first-year students an introduction to the analysis of complex data sets using bioinformatics and statistically reliable gas-exchange replicates. Assessment of the impact of this program revealed students to view the research as challenging, but confidence building as they take their first steps as biology majors. In addition, the direct mentorship of first-year students by upperclassmen and faculty was viewed positively by students. Ongoing assessments have revealed SAS participants to display a 15% increased persistence rate in STEM fields when compared to non-SAS biology majors.

## Introduction

The Students as Scholars (SAS) program was developed to welcome first-year students into college with a high level of mentoring, engagement, and community. The persistence of undergraduates in STEM majors is a focal point for improving the quality and diversity of graduates entering into scientific disciplines. Approximately 50% of students who declare a STEM major when entering college will change majors before graduation, with greater than 60% of underrepresented students changing majors (National Academies of Science Engineering and Medicine [NAS], 2017; Estrada et al., 2018). This “leaky pipeline” has been a historical focus of scholarly assessment and improvement of higher education in STEM, working to identify high impact practices that improve persistence in STEM majors. One focal point for this scholarship has been the potential benefit of undergraduate research experiences (UREs), which serve the central learning outcomes defined by the American Association of Colleges and Universities. These outcomes focus upon developing both intellectual and practical skills, applied learning, and both personal and social responsibility. A 2017 report by the National Academies of Science defines a URE program as one that provides students with authentic research experience, invested mentoring, a collaborative environment, opportunities to learn and interpret data, and improved skills in communicating results (National Academies of Science Engineering and Medicine [NAS], 2017). Noting the potential impact of URE programs, universities are investing more in authentic research experiences for STEM students. Programs include full-time summer undergraduate research programs, course-based undergraduate research experiences (CUREs), and part time participation in research during the academic semester. These opportunities vary widely with regard to student experience and expectations, but share a recognition that the active process of discovery is an authentic and compelling introduction to the heart of STEM disciplines.

Curriculum development to improve the experience of a STEM major will typically examine student engagement over 4 years: from initially being an observer of the research process to gradually becoming an active research scholar, displaying mature hypothesis formulation, experimental design, and professional engagement (Estrada et al., 2016; Wilson et al., 2018). Though challenging to independently assess, influential factors in student persistence include scientific efficacy, professional identification in community, close mentoring, and authentic research engagement early in the collegiate experience (Graham et al., 2013; Light and Micari, 2013; Estrada et al., 2018). UREs provide student experiences that improve persistence in the sciences, especially when provided during the first 2 years of college (Jones et al., 2010). Dating back to 1988, the Meyerhoff Scholars Program at the University of Maryland-Baltimore offers a carefully developed 4 year strategy for students pursuing research-oriented careers. The program includes research experience beginning in the first-year, partnered with early engagement of a scholarly community, summer research interships, advising, and sustained mentoring. The success of this program in improving student diversity on campus, and achieving strong STEM persistence with these populations has gained wide attention. In 2019, the “Meyerhoff Model” was used to develop similar programs at UC San Diego and UC Berkeley, funded through the Chan Zuckerberg Initiative. Though requiring a heavy institutional investment, including faculty time, these types of programs are successful in improving the persistence of under-represented populations of students.

The SAS biology program at Pepperdine University was developed in response to this evidence of the importance of student experience in the first-year and with the prior success of the Keck Scholars program for students majoring in diverse disciplines. Prior to these programs, student engagement in research during their first-year was limited, and often delayed until their junior year. This was especially the case for students requiring foundational coursework in microbiology, biochemistry, and cell biology. During their first-year in college, the first-year SAS program integrated student mentors, dedicated class time on specific foundational concepts, and applied a methodical approach to project design. With regard to the projects defined here, partnership between a first-year class and an upper division course in microbiology and plant physiological ecology was essential to project success. Students in the higher-level class brought skill sets in microbiological laboratory techniques, field portable gas-exchange, and data analysis which were essential to project success. In this manuscript, we focus upon SAS program content at Pepperdine and note that Whittier College was a partner in this work, though their method of enrolling students in first-year seminars was slightly different.

### Program Structure

**FIGURE 1:**
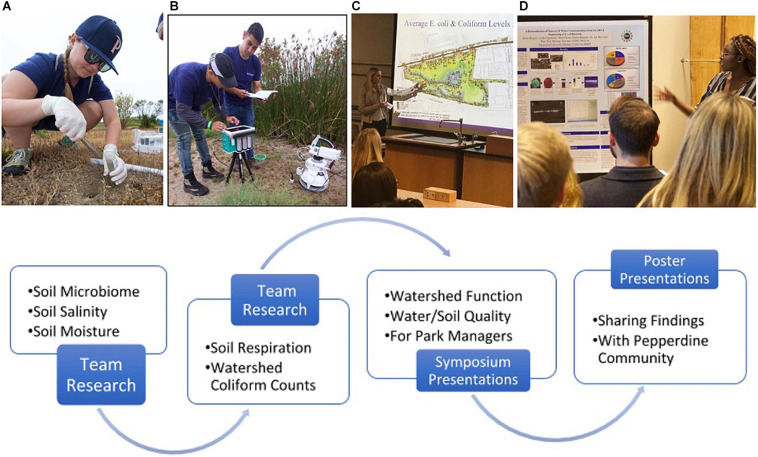
SAS-Program Activities: Pepperdine students **(A)** collecting soil samples for microbiome, salinity, and moisture analyses; **(B)** measuring soil respiration with a field-portable CO_2_ flux chamber and gas-exchange system; **(C)** giving symposium presentations on water and soil quality for environmental managers of coastal wetlands; and **(D)** sharing research findings in a final poster session for Pepperdine University’s community of learners, attracting over 120 participants. Photos by SD Davis.

The SAS program was developed within the context of first-year seminar classes, which integrate academic content with orientation topics, dialogue on collegiate best practices, intensive writing, and content on career exploration. Faculty teaching these classes met regularly during the school year to discuss class progress and best practices. Incoming biology majors enrolled in either a SAS course, or another first-year seminar class. It should be noted that all biology majors were encouraged to register for a SAS class as their first-year seminar. In the first year of the program, we had sufficient capacity (50–60 students) for all majors. In years 2 and 3, growth in the student population resulted in an inability of these classes to fully accommodate all majors, and thus some students enrolled in other first-year seminar classes. Comparisons in STEM persistence between the SAS and non-SAS students were recorded. We note that though capacity was a significant cause for biology majors enrolling in non-SAS classes, there is the potential that some students pre-selected against research, which would be predictive of a weak affinity for STEM disciplines. Classes met for 3 h of lecture time with an additional 3 h of time allotted for research in a teaching laboratory, greenhouse, and field sites. Biology students not taking a SAS first-year seminar class were enrolled in a biology colloquium class focused upon introducing the biology major to new students via topical content and group assignments. Early in the semester, all SAS courses introduced the scientific method and explored some research data from within their content area of study. An institutional volunteer day for community outreach and service was used by each SAS class to engage with resource managers of private, city, and state parks. The microbiological data collected ranged from assessing soil salinity, soil respiration, and plant water status in a local park struggling with salinity in the soil, to simple coliform counts within the Malibu watershed in areas experiencing vegetation dieback. The data collected was reported back to park managers during a symposium event hosted by the SAS classes (Figure 1). This integration of student training with public service was a helpful first step in building student teamwork and motivation for extending their work to full research projects. We also note student excitement that local park managers were appreciative of the data generated, information gleaned, and recommendations provided.

In the second half of the semester, students began to work in research groups, with an initial focus upon refining a research question. Groups met to discuss ideas, to read research papers, to engage with experienced research students from the biology major (class mentors), and ultimately to offer a research proposal to the class. This proposal was presented orally, and subsequently received feedback from the class, the research mentors, and from the professor. After feedback was received, students refined their project and submitted a final proposal for approval by the faculty member. Most projects required at least 6 weeks of data collection to address the research question. For microbiological projects, there was a range of work from assessing biodiversity in soils using ecoplate technology, quantification of coliform levels in local parks and lagoons, evaluation of soil respiration along a salinity gradient, and genomics-based assessment of soil microbial diversity. Each class had a timeline for concluding data collection and moving into analysis and preparation of a summative poster. Faculty recognized that student groups would be in varied stages of project completion, nonetheless, all groups developed a poster to tell the story of their project. The concluding poster session included all first-year biology students, administrators, faculty, upperclassmen from the biology major, and campus guests. Through NSF funding, students were able to submit a small grant proposal that could provide funding for further research the following summer. These proposals were competitively reviewed and awarded for a cohort of approximately 8 students/summer. We note that these SAS classes generated a significant number of students who continued their research engagement throughout their undergraduate training.

### Research Project Examples in Microbiology

As the SAS program matured, the faculty became more experienced with guiding first-year students to projects that were appropriate. The program goal was to develop projects that asked scholarly research questions and were practical in the context of first-year students and their available time. The importance of the student mentors was clearly realized during project design, students appreciated having experienced research students available to help them interpret the literature, and to develop research approaches. Faculty expressed the importance of hosting class projects with some overlapping techniques and focus, to simplify the work of research support and to build interest between research groups.

### Examination of Water Quality in the Malibu Community

Upon initial offering of this program, microbiological projects focused upon water quality testing and soil microbial diversity. The Malibu community has local parks, creeks, and marine estuaries that each have unique dynamics of watershed function including water contamination risks and management decisions. Malibu’s 6-acre Legacy Park was designed to reduce microbial contamination from local water flows using a naturalized stream channel and vegetated detention basin (Brager and Thorsen, 2011). Student groups met with the park managers to discuss the park design, and challenges with water management at the park. Students designed a project focused upon measuring coliform levels in part waterways and surge channels leading to the ocean as well as from groundwater at the site. Some samples were further evaluated by 16S rRNA gene sequencing to identify the microbes present. The project revealed a uniform distribution of coliforms throughout the park waterway, rather than a decline in concentration at the outflow point. Students interpreted this to be caused by higher than expected rates of water flow, and entry of water into the park from unanticipated locations. Though limited in scope, this provided the students with some experience with molecular biology techniques and genetic analysis. We note another project in which students were evaluating the filtering properties of marine estuaries. They measured coliform levels weekly along a local creek leading to an estuary adjacent to the Pacific Ocean. Interestingly, they identified a region of the creek that displayed a clear spike in coliform (including *E. coli*) counts in weekly tests. Investigation of that sampling by the students revealed a hidden encampment of homeless people adjacent to the creek and evidence of human waste along the creek margins. These microbiological projects provided technical engagement, insight into sampling methods, microbial culture experience, and an introduction to microbial identification via 16S rRNA gene sequencing.

### Analysis of Soil Microbiome and Respiration Through Cross-Course Collaborations With SAS First-Year Seminars

The faculty/students of one SAS class established a cross-course collaboration with a class focused upon genomics analysis which is titled, The Application of Genomic Strategies in Human and Microbial Diversity. This cross-course collaboration enabled research projects evaluating soil respiration and soil microbiome composition along a soil salinity gradient at Legacy Park (Figures 1A,B). Dr. Stephen Davis’ research group at Pepperdine has contributed significantly to the understanding of environmental stresses on chaparral vegetation (Venturas et al., 2016; Jacobsen et al., 2018). However, the effect of environmental stress on the interaction between the soil microbiome and Chaparral plant species represents an understudied research area and a unique opportunity to engage undergraduates in novel genomics and ecological research.

The objectives of this collaboration were threefold: (1) to introduce freshmen to the scientific process in preparation for engagement in an authentic research experience, (2) to introduce Pepperdine upperclassman to next-generation sequencing strategies and analysis, and (3) to provide scientific information for the wise management of Malibu’s Legacy Park and the park’s efficacy in soil water purification for the city of Malibu. We also wanted to determine the cause of selective plant mortality in Legacy Park for improved landscape management. Student’s tested the hypothesis that plant dieback patterns were associated with heterogeneity in soil salinity, soil microbial composition, and soil respiration. The collaborative project was especially impactful on beginning freshmen students who had just completed their first 3 weeks of college on the Malibu campus of Pepperdine University. First-year students experienced the scientific process of hypothesis formulation, experimental design, data reduction, interpretation of results, and presentation of their research findings before city managers of Legacy Park at a symposium and final poster session (Figures 1C,D).

### Analysis of Soil Salinity, Respiration, and Microbial Composition

The soil microbiome is a key contributor to the health of plants (Dubey et al., 2019). One way in which the microbiome directly influences surrounding plants is through the liberation of carbon dioxide through the process of respiration (Gougoulias et al., 2014). Consequently, shifts in the soil microbial environment could drastically alter atmospheric CO_2_ (Gougoulias et al., 2014). There is evidence that exposure to environmental stress alters the diversity of the soil microbial community (Hinojosa et al., 2016; Li et al., 2019; Whitman et al., 2019), however literature is lacking on the effect of environmental stress on Chaparral-microbiome interaction.

**FIGURE 2:**
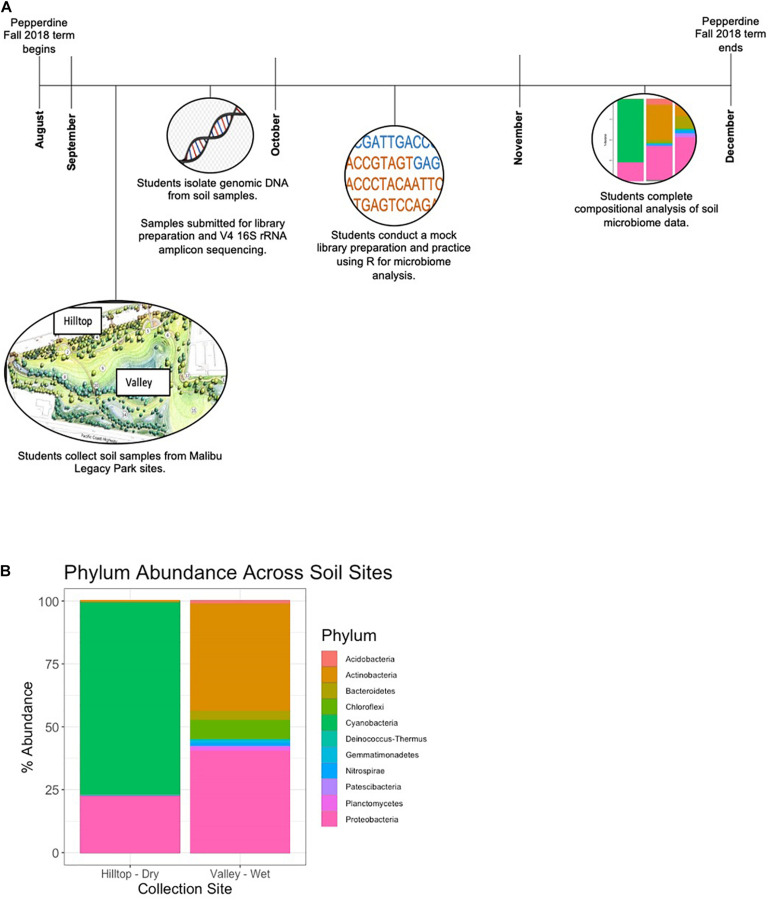
Soil microbiome analysis. **(A)** Timeline of the student-led soil microbiome sequencing project. **(B)** Relative bacterial phylum abundances across Legacy Park sites.

**FIGURE 3:**
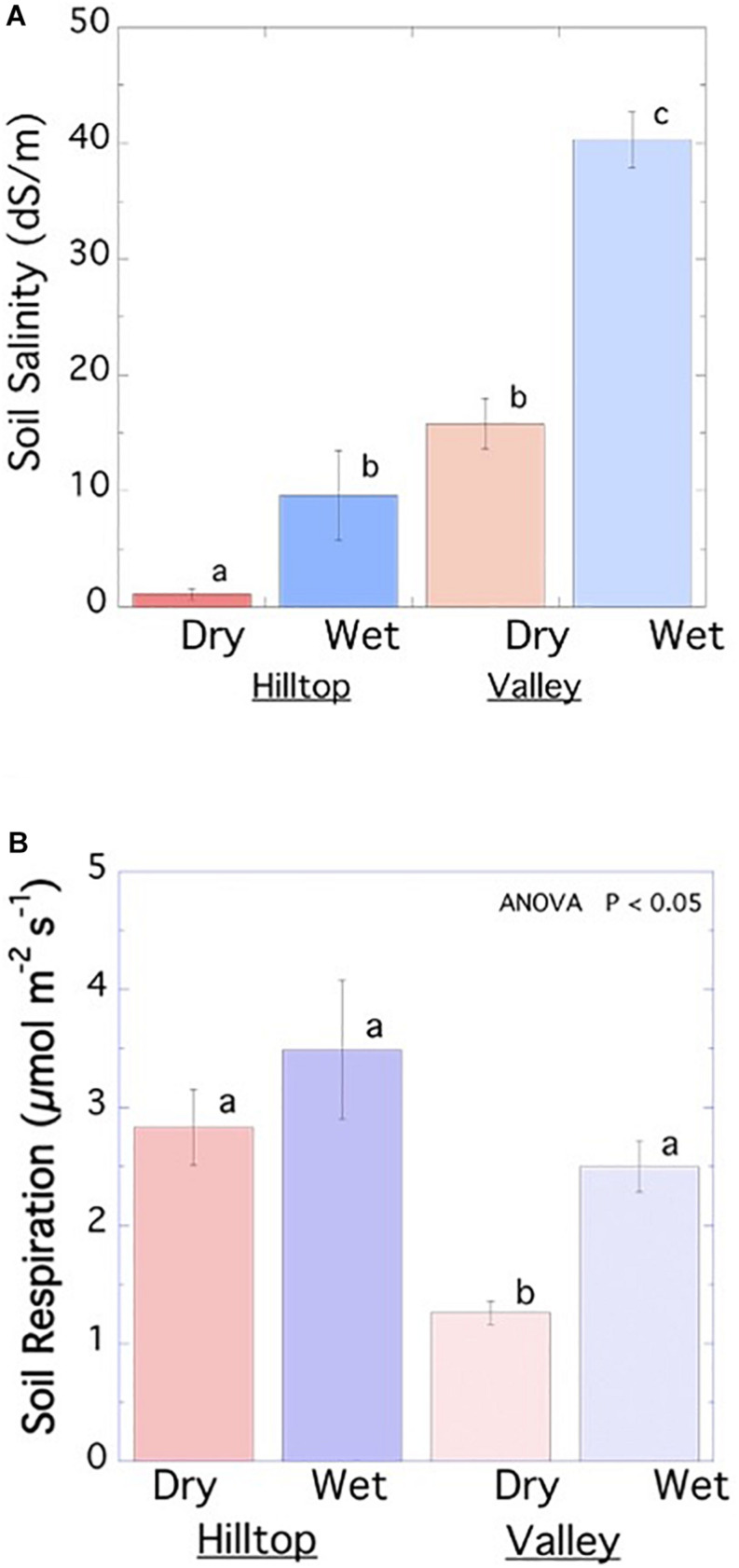
Analysis of soil **(A)** salinity and **(B)** respiration in Legacy Park at hilltop low soil salinity and high soil respiration sites compared to valley bottom sites experiencing high salinity and low respiration. Differences between letters on bar graphs indicate significant difference between treatments by one-way ANOVA followed by a Fisher’s least significant difference test at *P* < 0.05. Error bars on symbols represent ±1 SE, *n* = 3.

SAS students along with senior-level upperclassmen enrolled in the genomics course collected soil respiration data and samples for bacterial microbiome analysis from two sites in Malibu’s Legacy Park (Figure 2A). The two sites from which these data were collected represented a range of environments differing in dryness and salinity: the hilltop site was farthest from the creek bed, near a working irrigation system, while the valley site was near a moist creekbed. Respiration and salinity were measured in dry and wet soil from both sites, while microbiome composition was analyzed solely in the dry hilltop and wet valley sites (Figures 2, 3).

For analysis of microbiome composition, students first isolated metagenomic DNA from soil samples collected from each site using the QiaAmp DNA Stool MiniKit (Qiagen). An additional bead beating step was added to ensure proper isolation of both gram positive and gram-negative bacteria. Metagenomic DNA was then submitted to the UC Davis Host Microbe Systems Core for library preparation and targeted amplicon sequencing of the 16S ribosomal RNA gene. Samples were subjected to a nested PCR for library preparation prior to sequencing. Specifically, from the metagenomic DNA, the full length 16S rRNA gene was amplified prior to a second PCR during which the V4 region of the 16S gene was amplified, enhancing the ability to detect bacterial taxa in the samples. Prior to analysis by the students, samples were pre-processed using DADA2 and amplicon sequence variants (ASVs) were assigned taxonomy against the SILVA database (Callahan et al., 2016). Students then completed analysis of the soil microbiome using the R-based packages (Phyloseq and ggplot2), comparing relative abundances of bacteria at the phylum level (McMurdie and Holmes, 2013; Wickham, 2016; Figure 2). For relative abundance analysis, raw read counts are transformed to percent abundance, wherein the percent of each bacterium is calculated relative to the total number of reads per sample.

Students measured soil volumetric water content using time domain reflectometry, soil temperature with a thermistor, and soil salinity with a precision electrical conductivity sensor (Decagon 5TE sensor with ProCheck Meter, Decagon Devices, Inc., Pullman, WA). Electrical conductivity was converted to salinity in dS m^–1^ using a ratio of 10 g distilled water to 10 g soil in a 1:1 ratio and correlation to salinity measured by the saturated paste method. Students measured soil respiration using two separate soil CO_2_ flux chambers coupled to field portable gas-exchange systems (Li-6800, LiCor, Inc., Lincoln, NE). Students used three replicates at each sampling site after they installed 12 soil collars 3 days prior to initial sampling. Mean values were compared among treatments using a one-way ANOVA, followed by Fisher’s least significance tests, where appropriate (Figure 3B).

Soil salinity and respiration were measured in dry and wet soil from hilltop and valley sites in Legacy park (Figures 2A, 3). Soil salinity was highest at the wet valley site. This heightened salinity correlated with decreased soil respiration (relative to the hilltop sites) and increased bacterial diversity (Figures 2, 3). Bacterial community analysis from metagenomic soil DNA revealed increased abundances of Proteobacteria and Bacteroidetes in the valley site, taxa which have been previously associated with high soil salinity (Canfora et al., 2014). In the hilltop site, we note an increased abundance of Cyanobacteria. Soil biocrusts containing cyanobacteria have been reported to occur widely in undisturbed soils, including hilltops, of coastal sage scrub communities, similar to those of Legacy Park, in Malibu (Hernandez and Sandquist, 2011). One study reports a correlation between soil respiration rates and the abundance of certain classes of Proteobacteria (Li et al., 2011), suggesting this taxon could be contributing to the variation in soil respiration among the Legacy Park sites.

Though preliminary, the soil microbiome analysis provides key insight into the Legacy Park ecosystem. Coupled with the assessment of soil respiration and salinity, students are able to draw conclusions about the connectedness of the soil microbial community and the surrounding Chaparral environment. Further, this study highlights the feasibility of such collaborative research, conducted entirely by undergraduate students. Notably, soil respiration and salinity were analyzed by first-year undergraduates, guided primarily by their peer mentors.

In the future, additional samples can be collected from these sites in Legacy park to validate these findings. Other projects related to these soil collection sites include analysis of the soil mycobiome via sequencing of the internal transcribed spacer region (ITS sequencing) and/or quantification of specific bacterial and fungal species from soil using quantitative PCR. In addition, SAS students in the Davis and Stiemsma research groups have also begun using these techniques to study the effect of wildfire on soil microbial communities using samples collected after the Woolsey Fire of November 2018.

### Genomics Datasets as Teaching Tools in SAS and Other Life Sciences Courses

Interest in microbiome research continues to be received with great enthusiasm by the scientific community. Beyond the study of microbiome composition, health research and environmental ecology are examples of fields where the integration of microbiome analysis with other genomic strategies is utilized to assess host-microbiome interactions (Jansson and Hofmockel, 2018; Awany et al., 2019). The growing application of next generation sequencing has also significantly enhanced the roles of bioinformaticians and computational biologists in fields such as these, highlighting the need to engage students in these subjects early on in their undergraduate careers.

As represented by this cross-course collaboration with the SAS program at Pepperdine, studying the microbiome using next-generation sequencing is relatively easy to integrate into the classroom. The majority of computational tools (namely R) used to analyze microbiome sequencing data are open-source and free for users, decreasing the need to purchase expensive licenses for software or to implement additional laboratory fees to cover the cost of conducting these analyses. Further, the continued decrease in sequencing costs and streamlining of the wet and dry laboratory procedures to conduct microbiome analysis will significantly enhance the ability to conduct this applied research in the classroom.

Beyond our introduction of next-generation sequencing technology to and analysis of the human microbiome by this student group, we have also established lab modules that incorporate analysis of these datasets in other courses (e.g., Microbiology and Genetics). These lab modules serve to introduce students to the field of genomics and the use of R for microbial genomics analysis. Combined with more student-generated sequencing datasets, these modules will be key in expanding genomics in the SAS program. Further, the study of personal microbiomes in the classroom was reported to increase student engagement in genomics-based coursework (Weber et al., 2018) and represents one additional type of microbiome analysis that could be implemented into an SAS first-year seminar, among other life science courses at Pepperdine.

### Concluding Poster Session and Follow Through

At the conclusion of each SAS program, student research teams presented their findings at a reception and poster session. This was well attended by Pepperdine students, faculty, and administrators. Students regarded this event as intimidating, but an encouraging conclusion to their first semester of college. To date, these projects have resulted in 16 presentations at regional conferences, such as the Southern California Conference for Undergraduate Research, and 15 presentations at annual meetings of national scientific conferences such as the 113th Botanical Society of America meeting, 103rd Ecological Society of America meeting, and 58th American Society of Cell Biology meeting.

### Adapting SAS to the COVID-19 Environment

Notably, COVID-19 has dramatically altered the ability to conduct research at universities across the United States. Though wet-laboratory work may be challenging, we have highlighted ecological and genomics projects in this report that could be easily adapted to a virtual environment. Firstly, the collection of soil, water, and plant samples/assessments takes place outdoors, which limits aerosol transmission of microorganisms. Secondly, with regard to genomics research, once samples are collected, DNA can be isolated in a limited-personnel lab (maintaining social distances guidelines). Once DNA is isolated, the remaining analyses are computational, meaning the students can complete their analyses from anywhere with a sufficient internet connection. Pepperdine has also recently launched a secure server with access to R-studio for students enrolled in classes or research, further facilitating a virtual research environment.

### Assessment of the SAS Program Highlights Increased Retention of Students in STEM Disciplines

**FIGURE 4:**
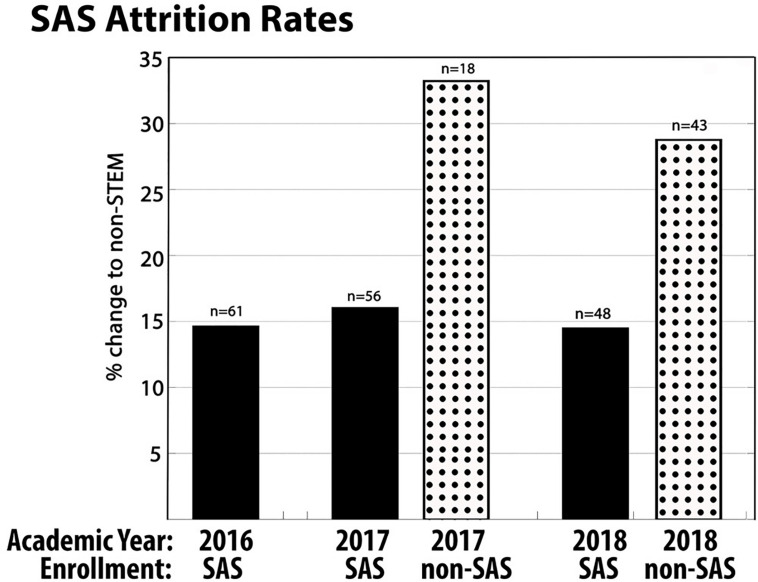
Retention of SAS and non-SAS students in STEM disciplines in 2016, 2017, and 2018. There are no data for non-SAS students in 2016 due to limited sample size. These data represent attrition rates for first-year biology students at Pepperdine University. These data were scored by declared major at the conclusion of the third semester of enrollment. All students assessed entered Pepperdine as biology majors.

The SAS program has been highly successful at Pepperdine. Throughout the program, students have participated in assessment through focus group discussions and pre/post program surveys. In the spring of 2020 the first cohort of SAS students were in our group of graduating seniors. Their feedback on exit surveys was highly supportive of the program, with several students noting the SAS program as critical in their decision to remain in sciences, or even at Pepperdine. We have collected data on student persistence in STEM disciplines since the establishment of the SAS program in 2016. Persistence in a STEM major was examined from student records at the conclusion of their third semester of Pepperdine enrollment. In the Pepperdine SAS programs of 2017 and 2018 our STEM discipline attrition rate averaged 15.3% while attrition of non-SAS biology students averaged 30.2% (Figure 4). Alternatively stated, we report 15% increased retention of students in STEM majors during their first year in college, reducing typical attrition by one-half. Also of note, Pepperdine assessed all first-year students in 2017, examining characteristics of student care, health, and connection at Pepperdine. As all first-year students participate in a first-year seminar class, we found this an opportunity to compare the Pepperdine SAS students to all other first-year students. When asked to rate the ease at which they have made friends during their first semester, 54% of the SAS cohort responded “very easy” while only 25% of students enrolled in other categories of first-year seminars responded similarly. When asked about the frequency of feeling loneliness, 9% of SAS students responded “often” or “all the time” contrasted with 19% of students in all other first-year seminars. When asked if their first-year seminar helped the student to transition into college, 40% of SAS students responded “a lot” while the average response from other first-year programs was 27%. This analysis of first-year seminars provided good support that this course structure was providing community to our incoming students, and an improved integration into the campus environment.

**FIGURE 5:**
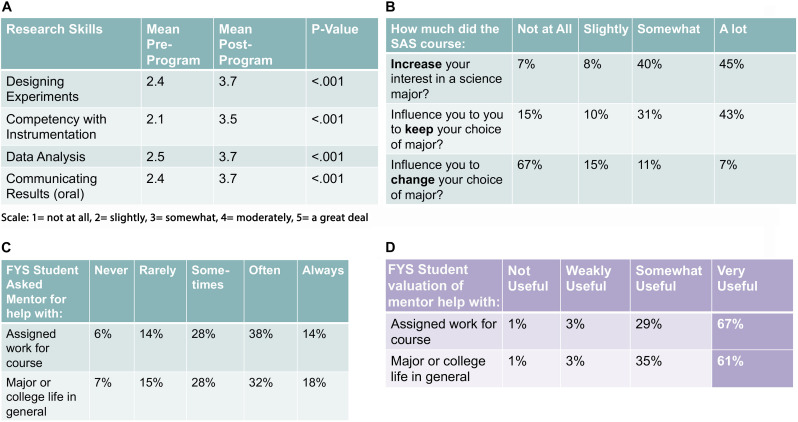
Exemplar SAS course assessment data generated from student surveys and focus groups. Data in panel **(A)** represents a comparison of student assessments of efficacy in the four dimensions of research ability before and after program participation. These paired measures were analyzed by *t*-test with statistical significance assigned to *p* < 0.05. Panels **(B–D)** offer representative data from program surveys offered upon conclusion of the program. Panel **(B)** displays student reporting of program influence upon interest and retention in a science major. Panels **(C)** and **(D)** reveal frequency and valuation of student access to a faculty mentor. All of the data shown represents feedback from SAS students in the 2017 program cycle with 90% student participation in assessment (*n* = 110). Pepperdine and Whitter college students are equally represented in these data.

An annual process of SAS assessment for both Pepperdine and Whittier College students was managed by the WestEd educational consulting group, and the data were reported in combined format. We have provided some key points of information from that assessment, using data from the 2017 SAS groups at both institutions. The data shown are consistent with assessments from other years. Please note that full participation in this assessment was a requirement of program participation. Student self-efficacy in research was assessed on four dimensions of research ability; (1) designing experiments, (2) competency with instrumentation, (3) data analysis, and (4) communicating results). When comparing pre/post program participation, student self-efficacy increased significantly from an average of 2.4 prior to completing an SAS course to 3.7 after completing an SAS course (Figure 5A). These self-assessments suggest an overall level of increased confidence in conducting STEM research amongst SAS scholars. Questions focusing upon student attitudes about their science major examined factors that are likely to predict persistence. The majority of SAS students (85%) report that their involvement in an SAS course increased their interest in a science major either “somewhat’ or “a lot” (Figure 5B). Also, 73% of SAS students stated that their enrollment in an SAS course influenced them to keep their choice of major. These two data points address a key goal of the program, to improve student persistence through the critical first-years of college. Beyond persistence in STEM disciplines, participation in the SAS program was key in enhancing student interest and efficacy in research. Specifically, 85% of students reported an interest in pursuing other research opportunities during their undergraduate career and 73% of SAS students report that they are confident they will present their research at a research conference in the near future. The influence of student mentors and faculty mentors in their academic success and transition into college life were perceived as extremely valuable (Figures 5C,D). Collectively, these data highlight the importance of the SAS program in retaining STEM majors and enhancing student learning at the undergraduate level. Students who participated in the SAS program engaged more with their peers, gained confidence in their ability to apply life science content through research, and showed greater likelihood of continuing on in STEM for the duration of their undergraduate careers at Pepperdine.

## Conclusion

The Students as Scholars program was designed in response to the research literature on undergraduate engagement and its impact upon the student success. Implementation of the program required the partnership of committed faculty willing to engage significant time in the mentoring of first-year students. In addition, the program required an infusion of funding from the National Science Foundation to support the cost of research supplies, stipends for student mentors, and research fellowships for students wishing to continue their research. A sustainable program will need to identify program components that accomplish key characteristics of the program, but within the available time from busy faculty, and within the budget available from the host institution. In our experience, this program provided clear improvement of student engagement, persistence, and satisfaction with their science major. The integration of scholarly work in microbiology was particularly appropriate due to the range of important questions that first-year students can experimentally evaluate, and the cohesion of traditional microbiological research with more advanced microbiome analyses linked to ecosystem services of fertile soil and clean water. Collaboration between a first-year seminar and an advanced elective course was surprisingly effective. The more experienced students were energized by the partnership and proud to display their expertise and to train younger students. Similarly, student mentors established healthy relationships with their class and played an important role in project design and execution. As faculty became convinced of the benefits for students, recruiting participating faculty became easier. We are working to modify the program to implement the program without external funding, and to expand the program beyond students majoring in biology. The SAS program has provided clear support for institutional investment in the first semester of college to ensure students arriving with a disciplinary interest have the opportunity to truly experience the merits and community available to them in that particular STEM discipline.

## Data Availability Statement

The datasets presented in this study can be found in online repositories. The names of the repository/repositories and accession number(s) can be found below: https://www.ncbi.nlm.nih.gov/, PRJNA649789.

## Ethics Statement

Written informed consent was obtained from the individual(s) for the publication of any potentially identifiable images or data included in this article.

## Author Contributions

All authors contributed to the data analysis and writing of this manuscript.

## Conflict of Interest

The authors declare that the research was conducted in the absence of any commercial or financial relationships that could be construed as a potential conflict of interest.
